# Rationale and design of a randomised controlled trial investigating the effect of multidisciplinary nutritional rehabilitation for patients treated for head and neck cancer (the NUTRI-HAB trial)

**DOI:** 10.1186/s12937-020-00539-7

**Published:** 2020-03-17

**Authors:** Marianne Boll Kristensen, Irene Wessel, Anne Marie Beck, Karin B. Dieperink, Tina Broby Mikkelsen, Jens-Jakob Kjer Møller, Ann-Dorthe Zwisler

**Affiliations:** 1 REHPA, The Danish Knowledge Centre for Rehabilitation and Palliative Care, Department of Clinical Research, University of Southern Denmark, Odense, Denmark; Odense University Hospital, Vestergade 17, Nyborg, DK-5800 Denmark; 2 Department of Nursing and Nutrition, University College Copenhagen, Sigurdsgade 26, DK-2200 Copenhagen N, Denmark; 3 OPEN, Odense Patient data Explorative Network, Odense University Hospital, J.B. Winsløws Vej 9A, DK-5000 Odense C, Denmark; 4 Department of Otorhinolaryngology, Head and Neck Surgery & Audiology, Rigshospitalet, Blegdamsvej 9, DK-2100 Copenhagen Ø, Denmark; 5 Dietetics and Clinical Nutrition Research Unit, Herlev and Gentofte Hospital, Borgmester Ib Juuls Vej 50, 4, DK- 2730 Herlev, Denmark; 6 Research Unit of Oncology, Department of Oncology, Odense University Hospital, Sdr. Boulevard 29, 5000 Odense C, Denmark; 7 Department of Clinical Research, University of Southern Denmark, J.B. Winsløws Vej 19.3, 5000 Odense C, Denmark

**Keywords:** Head and neck cancer, Rehabilitation, Survivorship, Eating problems, Quality of life, Assessment of rehabilitation needs, Nutritional assessment, Nutrition screening

## Abstract

**Background:**

Eating problems frequently affect quality of life and physical, psychological and social function in patients treated for head and neck cancer (HNC). Residential rehabilitation programmes may ameliorate these adverse effects but are not indicated for all individuals. Systematic assessment of rehabilitation needs may optimise the use of resources while ensuring referral to rehabilitation for those in need. Yet, evidence lacks on which nutrition screening and assessment tools to use. The trial objectives are: 1) To test the effect of a multidisciplinary residential nutritional rehabilitation programme compared to standard care on the primary outcome body weight and secondary outcomes health-related quality of life, physical function and symptoms of anxiety and depression in patients curatively treated for HNC and 2) To test for correlations between participants’ development in outcome scores during their participation in the programme and their baseline scores in Nutritional Risk Screening 2002 (NRS 2002), the Scored Patient-Generated Subjective Global Assessment Short Form (PG-SGA SF), and M. D. Anderson Dysphagia Inventory (MDADI) and to assess sensitivity, specificity and predictive values of the three tools in relation to a clinically relevant improvement in outcome scores.

**Methods:**

In a randomised controlled trial, 72 patients treated for HNC recruited through a nationwide survey will be randomised to a multidisciplinary residential nutritional rehabilitation programme or to a wait-list control group. Data are collected at baseline, three and six months. Primary outcome is change in body weight, and secondary outcomes include changes in quality of life, physical function and symptoms of anxiety and depression. Potential correlations between intervention effect and baseline scores in NRS 2002, PG-SGA-SF and MDADI will be tested, and sensitivity, specificity and predictive values of the three tools in relation to a clinically relevant improvement in outcome scores will be assessed.

**Discussion:**

This is the first randomised controlled trial to test the effect of a multidisciplinary residential nutritional rehabilitation programme in patients treated for HNC. Recruitment through a nationwide survey gives a unique possibility to describe the trial population and to identify potential selection bias. As the trial will explore the potential of different nutrition screening and assessment tools in the assessment of rehabilitation needs in patients treated for HNC, the trial will create knowledge about how selection and prioritisation of nutritional rehabilitation aimed at patients treated for HNC should be offered. The results may contribute to a better organisation and use of existing resources in benefit of patients treated for HNC.

**Trial registration:**

The trial is registered by The Danish Data Protection Agency (registration 2012-58-0018, approval number 18/14847) and the Regional Committees on Health Research Ethics for Southern Denmark (journal number 20182000–165). ClinicalTrials.gov Identifier: NCT03909256. Registered April 9, 2019.

## Background

The incidence of head and neck cancer (HNC) has increased to approximately 900.000 new cases worldwide in 2018 [1, 2]. With a simultaneous increase in the relative survival [3], the population of patients treated for HNC is increasing.

Many patients treated for HNC feel unprepared for the life that awaits them after cancer treatment [4–7] when eating problems and other late effects may persist for years or even become chronic [8]. These include dysphagia (swallowing difficulties), xerostomia (dry mouth), dysgeusia (taste disturbances), and trismus (reduced mouth opening) [8]. The negative effects of eating problems on quality of life (QOL) and everyday life in patients treated for HNC have been documented in quantitative [8–13] and qualitative [5, 14–19] studies. Based on existing studies [4–7, 18, 20, 21] it is suggested that appropriate rehabilitation services can strengthen the patient’s ability to cope with eating problems and thereby reduce the negative consequences. Yet, unmet rehabilitation needs are widely documented in this population [4, 5, 7, 16, 22, 23].

A frequent strategy for patients treated for HNC to cope with eating problems is the trial-and-error approach [4, 6, 16, 20] with continuous experiments to find tolerated foods as this varies over time. The process may be complicated by fear of choking [4, 15, 19] and feelings of defeat when experiments are unsuccessful [4, 16]. Residential group based rehabilitation programmes, where the daily meals are part of the intervention, may be particularly effective to support patients treated for HNC in this coping process as they can provide a safe environment to practice eating skills [4, 21]. High participant satisfaction and improvements in QOL scales were seen among patients treated for HNC participating in a pilot study testing a 1-week residential psychoeducational programme [21]. In another pilot study, qualitative data showed that patients treated for HNC benefitted from participating in a multidisciplinary residential nutritional rehabilitation programme [4]. Unpublished quantitative data from the latter pilot study (included in Additional file 1) showed significant improvements in body weight and several QOL scales at 3-month follow-up. With no control group in the pilot study, the results should be tested in a randomised controlled trial on the effect of the multidisciplinary residential nutritional rehabilitation programme.

The increasing population of patients treated for HNC may present a challenge to existing health care systems through increased rehabilitation costs. Residential rehabilitation programmes and other specialised rehabilitation services aimed at eating problems may be costly, and may not be indicated for all patients treated for HNC. Systematic screening and/or assessment of rehabilitation needs in patients treated for HNC may optimise the use of existing resources while ensuring referral to appropriate rehabilitation services for those in need.

The European Society for Clinical Nutrition and Metabolism recommends that nutritional screening is performed at cancer diagnosis and repeated regularly depending on the stability of the clinical situation [24]. Several tools have been developed to screen and assess nutritional risk, nutritional status and nutrition impact symptoms [24–28]. Nutritional Risk Screening 2002 (NRS 2002), [26] is validated to identify patients, regardless of their diagnosis, who will benefit from nutritional intervention. Yet, to our knowledge no studies have validated NRS 2002 in patients treated for HNC after treatment, and even for patients with HNC prior to treatment, it has been suggested to use a modified version with a different cut-off value [29]. Furthermore, NRS 2002 only assesses dietary intake as the consumed amount in relation to requirements [26]. It does not assess nutrition impact symptoms, which would be highly relevant in this population. The Scored Patient-Generated Subjective Global Assessment Short Form (PG-SGA SF) includes information on nutrition impact symptoms and changes in dietary intake (amount or consistency) in the assessment of nutritional risk and nutritional deficit [27], but no validation studies have been carried out in patients after treatment for HNC. The M. D. Anderson Dysphagia Inventory (MDADI) [28] is developed to assess dysphagia-specific QOL in patients with HNC. But so far, no clinical studies have investigated associations between MDADI score and intervention effect. Hence, the evidence is scarce on the three tools’ ability to identify patients treated for HNC who will benefit from posttreatment nutritional rehabilitation.

### Trial objectives

The objectives of the trial are:

- To test the effect of a multidisciplinary residential nutritional rehabilitation programme compared to standard care on the primary outcome body weight and secondary outcomes health-related QOL, physical function and symptoms of anxiety and depression in patients curatively treated for HNC
- To test for correlations between participants’ development in outcome scores during their participation in the programme and their baseline scores in NRS 2002 [26], PG-SGA SF [27], and MDADI [28] and to assess sensitivity, specificity and predictive values of the three tools in relation to a clinically relevant improvement in outcome scores

## Methods

### Trial design

**Fig. 1:**
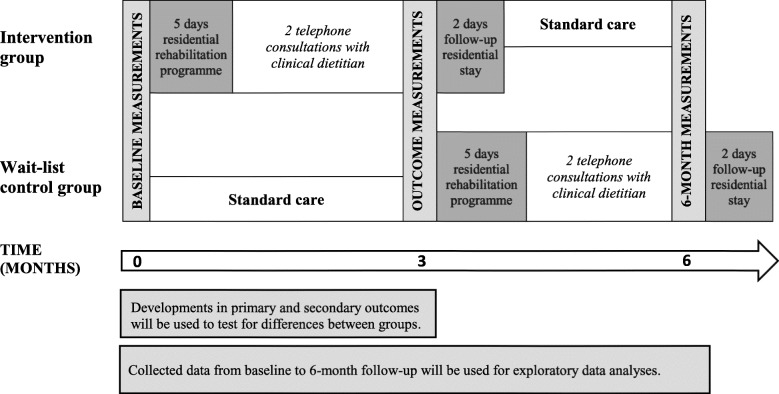
Timeline of the NUTRI-HAB trial

The trial is a randomised controlled trial with recruitment through a nationwide survey. Participants will be randomised into either an intervention group or a wait-list control group. Data will be collected at baseline, at three, and at six months (Fig. 1).

Differences between groups at 3-month follow-up will be tested to assess the effect of the intervention. Exploratory analyses will be based on all data collected from baseline to 6-month follow-up. They will include analyses of the long-term effect of the intervention and of whether the selected nutrition screening tools are labile and able to reflect changes over time.

The SPIRIT (Standard Protocol Items for Randomized Trials) 2013 [30, 31] statement, the CONSORT (Consolidated Standards of Reporting Trials) extension for reporting trials of nonpharmacologic treatments [32] and the TIDieR (template for intervention description and replication) [33] checklist and guide have been used as guidelines for developing the trial protocol. The SPIRIT checklist is included in Additional file 2, and descriptions of all physical and informational materials used in the trial and how to assess these are included in Additional file 3.

### Setting

The trial will be carried out at REHPA, the Danish Knowledge Centre for Rehabilitation and Palliative Care in Nyborg, Denmark, between May 2019 and December 2019.

In Denmark, cancer treatment and rehabilitation are funded by government taxes and free of charge for patients. While cancer treatment and rehabilitation services during treatment are offered at the hospitals, posttreatment rehabilitation is primarily a municipal responsibility [34]. Denmark comprise 98 municipalities with great variation between their rehabilitation services [35, 36]. Only 17 Danish municipalities offered diagnosis specific rehabilitation services for patients treated for HNC in 2017 [36]. Hence, the level of rehabilitation that participants have received prior to their participation in the trial may vary, and information on which rehabilitation services participants have been offered and participated in will be registered.

### Participants

Participants will be recruited among respondents of a nationwide survey on late effects and health-related QOL in Danish patients treated for HNC 1–5 years following radiation therapy. The survey population was identified through The Danish Head and Neck Cancer Group’s (DAHANCA) national clinical quality database [37].

The survey was distributed in March 2019. Patients treated for HNC will be eligible for participation in the trial if they meet the following inclusion criteria:

Register-based information:

- Have been diagnosed with cancer of the larynx, pharynx, or oral cavity
- Have completed curatively intended treatment with radiation therapy 1–5 years before survey distribution (1st of March 2014 to 28th of February 2018)
- Are aged ≥18 years

Self-reported information collected through the survey:

- Have no active HNC or any other active cancer at the time for completion of the survey
- Are self-reliant. Survey respondents are defined as self-reliant if they answered “Not at all” on the question “Do you need help with eating, dressing, washing yourself or using the toilet?” in The European Organization for Research and Treatment of Cancer’s (EORTC) QLQ-C30 questionnaire [38] on health-related QOL
- Are able to speak and understand Danish
- Have confirmed that they are interested in participating in a multidisciplinary residential nutritional rehabilitation programme at specific dates and given their permission to be contacted with further information. This inclusion criterion has been established to obtain permission and contact details for telephone contact and to narrow down the population for inclusion since the nationwide survey was distributed to almost 2000 individuals. By giving survey respondents the possibility to opt out for further contact regarding the trial, we reduce the number of inquiries to each respondent.

Potential recurrence of cancer during the trial will not lead to exclusion of participants. In the event of cancer recurrence in one or more participants, sensitivity analyses will be made to investigate whether this affects the trial results.

### Intervention

The intervention is a multidisciplinary residential nutritional rehabilitation programme with a primary focus on the physical, psychological and social aspects of eating problems after treatment for HNC. The programme will comprise five days initial residential stay and two days follow-up residential stay after three months (Fig. 1). The rehabilitation centre has developed a core programme model through available evidence and more than 10 years’ experience in offering multidisciplinary residential rehabilitation programmes for heterogeneous groups of patients with cancer [39, 40]. To meet the specific rehabilitation needs of patients treated for HNC, the core programme was further developed through available evidence, patient involvement and a pilot study including 40 patients treated for HNC [4]. Components of the rehabilitation centre’s core programme will be included even though they are not specifically aimed at eating problems. Yet, these activities have shown to be relevant and beneficial to other groups of patients with cancer [39–41]. The programme consists of group sessions with patient education and a few individual activities. The content of these sessions and activities are shown in Table 1 while a detailed schedule of the programme is shown in Additional file 4.

**Table 1:** Patient education sessions in the multidisciplinary residential nutritional rehabilitation programme [42] in the NUTRI-HAB trial

SESSION	DURATION (minutes)	CONTENT	SESSION LED BY
Welcome session with presentation of the programme^a,d^	30	The aim of the welcome session is to make participants feel safe and comfortable in the environment in which they will be spending the next five days. This may contribute to increased motivation and willingness to participate actively throughout the programme [43, 44].	Course leader^f^ and clinical dietitian
Presentation round^a,d^	60	In the presentation round, participants will share information about their background and cancer diagnosis, and they will be encouraged to use selected picture cards to narratively describe their expectations and desired outcomes of participation. The aim of the session is to enhance group formation and to establish a sense of community among participants since this may facilitate patient empowerment [45]. Central staff members (course leader, physician, clinical dietitian, and evening hostess) participate.	Course leader
Social activity^a,d^	60	A social activity including music and movement will be scheduled on the first evening of the programme to support group formation and candidness among participants.	Music therapist
Theoretical session on eating problems^a,d^	105	The session will include dietary advice to manage different nutrition impact symptoms e.g. choice of foods, texture and flavour modification [24, 46]. Exchange of experiences between participants will be encouraged.	Clinical dietitian
Individual dietary counselling^c,d,e^	30 (20 at follow-up)	In the individual dietary counselling, dietary advice will be tailored to the individual participant [24].	Clinical dietitian
Practical kitchen workshop^b,d^	180	In the practical kitchen workshop, participants will prepare foods of different textures and flavours, and take-home recipes will be handed out. The aim of the workshop is to inspire and put theory into practice [4], and practical kitchen sessions have supported dietary changes in studies with other types of cancer survivors [47, 48].	Clinical dietitian
Swallowing exercises^b,d^	90	Participants will be instructed in different swallowing exercises and exercises for jaw and tongue mobility, since these types of exercises may reduce dysphagia and trismus [46, 49]. Participants will receive an exercise manual and a training diary, and will be encouraged to continue doing the exercises, when they come home.	Occupational therapist
Dental problems and oral hygiene^b,d^	75	Dental problems are frequent after treatment for head and neck cancer [22]. The session will include information on how to maintain good oral hygiene and on dental reimbursement rules in relation to cancer treatment.	Dental hygienist
Physical activity^a,d,e^	75	Physical activity may contribute to ameliorate late effects associated with decreased physical function in cancer survivors [50–53]. In the physical activity sessions, participants will be introduced to different kinds of physical activity that they can do at home e.g. balance or resistance training exercises. Exercises will be adjusted to the participants’ training level.	Physiotherapist
Yoga^b,d^	60	Yoga may contribute to improve quality of life and to reduce fatigue and symptoms of distress and anxiety in cancer survivors [54, 55]. The yoga session will be based on principles from Hatha yoga and Physioflow yoga. Special attention will be given to exercises aimed at releasing tensions in the head and neck area.	Physiotherapist certified as yoga instructor
Psychological reactions to cancer^b,d^	150	The session will be based on a psychoeducational approach [56, 57] and will aim at supporting participants’ coping of everyday life after cancer. The session will comprise psychologist’s presentation of frequent psychological reactions to cancer and discussions in small groups.	Psychologist
The existential dimension of rehabilitation^a,d^	90	The session is a group conversation on questions of existential and spiritual character that often follow the diagnosis of a life-threatening disease [58, 59].	Priest
Massage therapy^c,d^	45	Massage therapy may contribute to short term reduction of pain and anxiety even though the level of evidence is very low [60]. Each participant will receive 45 min of relaxing massage therapy and will have to choose between a full body relaxing massage or special attention given to a certain area e.g. tensions in the neck.	Massage therapist
Vocational counselling^b,d^	75	Optional session. Vocational counselling session will aim to support return-to-work processes and hence participants functioning in accordance with the World Health Organization’s International Classification of Functioning, Disability and Health (ICF) [61]. The session will include information on rights and obligations according to Danish legislation.	Social worker
Fatigue and sleep problems^b,d^	75	Optional session on reasons for and management of cancer-related fatigue [52, 62] and sleep problems [63, 64].	Nurse
Motivation, goal setting and action plans^a,d^	100	Based on principles of motivational interviewing [65], the session will allow participants to reflect on, how they will implement new inspiration and knowledge gained through the programme, when returning back home.	Course leader
Intimacy and sexuality^b,e^	90	Optional session. Based on the PLISSIT model [66], the session will address how sexuality and intimacy can be affected by cancer and cancer treatment [67, 68] and provide advice for management of potential challenges. Participants will be divided into groups by gender for the session.	Sexologist
Meaning and values in life^b,e^	90	Optional session. Based on principles of acceptance and commitment therapy [69], the session will aim to support participants in re-establishing meaning in life through reflections on values and sources to meaning in lif e[70, 71].	Psychologist
Individual counselling^c,d,e^	30–45	Individual counselling with relevant health professionals (e.g. speech pathologist, physician) will scheduled depending on participants’ needs.	Depending on need

^a^ Group session with a maximum of 20 participants; ^b^ Group session with a maximum of 10 participants; ^c^ Individual session; ^d^ Session is offered at the initial five days residential stay; ^e^ Session is offered at the two days follow-up residential stay after three months; ^f^ Course leader (nurse, physiotherapist or social worker) coordinates all activities during the week and is the participants’ primary contact person

Rehabilitation Centre Dallund and REHPA, The Danish Knowledge Centre for Rehabilitation and Palliative Care have developed the core model for the residential group-based rehabilitation programme as a best practice patient-centred rehabilitation model for heterogeneous groups of cancer survivors. The rationale, evidence base and content of the model and the specific activities are described in details elsewhere [42]. The core model has been adjusted to meet the rehabilitation needs of the population in the NUTRI-HAB trial

As described in the background section, patients treated for HNC frequently use the trial-and-error approach [4, 6, 16, 20] to cope with their eating problems. The programme aims to support participants in this coping process in various ways. Participants will stay at the premises during the residential stays and all meals will be served in the dining room and break areas. Meals will be served as self-service buffets, and foods of different textures and flavours will be served to inspire and to allow participants to experiment. The menu plan for the entire residential stay will be presented on the first day and will be available in the dining room. If a participant has specific dietary preferences or requirements that are not met in the menu plan for a given meal, the kitchen staff will find alternatives together with the participant. The meals are furthermore intended as social training since eating problems often lead to social withdrawal [4, 15, 17, 20]. Individual counselling sessions with relevant professionals (e.g. speech pathologist or physician) will be scheduled depending on the individual participant’s needs assessed by baseline questionnaires and outcome data.

Between the initial stay and the two days follow-up, participants will have two telephone consultations with a clinical dietitian. These will be scheduled in week 4 and week 8. The aims of these consultations are to follow up on topics addressed in the individual consultation at the residential stay, to answer potential questions that have emerged, and to encourage the participant to continue with any activities or changes that they planned to implement after the residential stay.

The programme will be free of charge for participants and an additional offer to existing rehabilitation services. Participants will be asked to fill out an evaluation form in which they will evaluate the overall residential stay, the different sessions and indicate whether they participated in the specific session.

### Wait-list control group

Between baseline and 3-month follow-up, the wait-list control group will receive no intervention other than standard care. Since participants will be from all over the country, standard care may vary. Participants will not be restricted from participating in other rehabilitation services during the trial period. After 3-month follow-up, participants in the wait-list control group will be offered participation in the multidisciplinary residential nutritional rehabilitation programme.

### Inclusion and randomisation

**Fig. 2:**
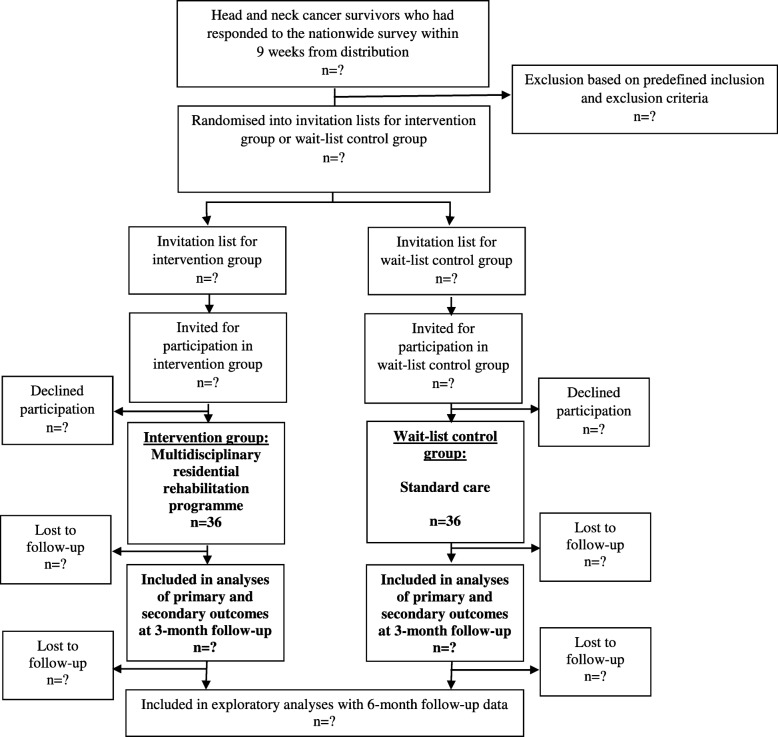
Flow chart of the NUTRI-HAB trial

Figure 2 shows the flow of the inclusion and randomisation process. Individuals who have responded to the nationwide survey within nine weeks from survey distribution and who meet the inclusion criteria will be randomised into invitation lists for intervention group or wait-list control group. The allocation ratio will be 1:1, and allocated individuals will be placed in random order on the numbered invitation list. Four residential rehabilitation programmes are scheduled, and each has a maximum capacity of 20 participants. Hence, a maximum of 40 participants in each group can be included. The first 40 individuals on each invitation list will receive further information about the trial and be invited to participate. Invitations will be sent electronically to e-Boks, a secure digital mailbox linked to the individual’s civil registration number. In Denmark, it is mandatory to have e-Boks unless a citizen applies for exemption. Individuals without e-Boks will receive the invitation through postal mail. If the invitation is declined, the next person on the given invitation list will be invited. If invited individuals do not respond, they will be contacted by telephone.

Randomisation will be stratified by need for rehabilitation services measured by the REHPA scale adapted from the National Comprehensive Cancer Network® Distress Thermometer [72]. On the REHPA Scale, participants indicate how close or how far they are from living the life they want after or in spite of their disease. A higher score indicates greater rehabilitation needs. The REHPA Scale was included in the nationwide survey. A certain score on the REHPA scale is not an inclusion criteria for the present trial, but randomisation will be stratified to ensure similar proportions of individuals with a score of ≥3 across the invitation lists. Participants will be randomised in STATA/IC 15.1 by a blinded researcher (TBM) who is not involved in the trial intervention or assessment of outcomes.

### Outcome measures and data collection

Outcome measures will be collected at entry, at three, and at six months (Fig. 1). Baseline measurements of the wait-list control group and 6-month follow-up measurements of the intervention group will be performed in one of three regional outpatient clinics depending on the participant’s place of residence. All other measurements will be performed at the rehabilitation centre. Data collected at different time points are shown in Table 2.

**Table 2:** Data collection at the different time points in the NUTRI-HAB trial

	TIMEPOINT		
	Baseline	3-month follow-up	6-month follow-up
**DEMOGRAPHIC DATA**			
**Register-based information**			
- Age	X		
- Gender	X		
- Cancer diagnosis	X		
- Time interval since treatment	X		
**Self-reported information**			
- Civil status	X		
- Educational level	X		
- Occupational status	X		
- Current cancer status	(X)	(X)	(X)
- Participation in other rehabilitation services	(X)	(X)	(X)
**NUTRITIONAL RISK AND PRESENCE OF NUTRITION IMPACT SYMPTOMS**			
- NRS 2002	X	(X)	(X)
- PG-SGA SF	X	(X)	(X)
- MDADI	X	(X)	(X)
**REHABILITATION NEEDS MEASURED BY THE REHPA SCALE**			
	(X)	(X)	(X)
**PRIMARY OUTCOME**			
- Body weight	X	X	(X)
**SECONDARY OUTCOMES**			
**Patient-reported outcome measures**			
*Quality of life*			
- EQ-5D-5 L	X	X	(X)
- EORTC QLQ-C30	X	X	(X)
- EORTC QLQ-H&N35	X	X	(X)
*Symptoms of anxiety and depression*			
- HADS	X	X	(X)
**Physical measurements and tests**^**a**^			
- Body mass index	X	X	(X)
- Maximal mouth opening	X	X	(X)
- Hand grip strength	X	X	(X)
- 30-second chair stand test	X	X	(X)
- 6-minute walk test	X	X	(X)

X: Data will be collected for primary analyses, (X): Data will be collected for exploratory analyses

^a^The physical performance tests will be made in a standardised order as follows: 30-second chair stand test, hand grip strength, and 6-minute walk test

EORTC: European Organization for Research and Treatment of Cancer, HADS: Hospital Anxiety and Depression Scale, NRS 2002: Nutritional Risk Screening 2002, PG-SGA SF: The Scored Patient Generated Subjective Global Assessment Short Form, MDADI: M. D. Anderson Dysphagia Inventory

Trained health professionals will perform all physical measurements and tests following strict protocols. Patient reported outcome measures and other patient reported data will be collected through electronic questionnaires distributed through Research Electronic Data Capture (REDCap) [73] to participants’ e-Boks one week before the scheduled physical measurements. Participants without e-Boks will be asked to fill out the questionnaire on a computer on the location of the measurement. Participants who are not confident in filling out the questionnaires electronically will fill out a paper-based questionnaire.

Data from paper-based questionnaires and results from physical measurements and tests will be entered in REDCap by one researcher, and the entered data will be double-checked by a second researcher.

#### Demographic data

##### Register-based data

Information on age, gender, cancer diagnosis and treatment was obtained from DAHANCA’s national clinical quality database [37] before the nationwide survey was sent out.

##### Self-reported data

Questions on civil status, educational level, occupational status, will be included in the electronic questionnaire at baseline. Questions on current cancer status and participation in other rehabilitation services prior to baseline was included in the nationwide survey. At 3-month and 6-month follow-up information on this will be collected through individual counselling sessions with the clinical dietitian.

#### Nutritional risk and presence of nutrition impact symptoms at entry of the rehabilitation programme

##### Nutritional risk screening 2002

The NRS 2002 has been developed and validated to identify admitted patients who will benefit from nutritional intervention [26]. Screening with NRS 2002 comprises a primary screening and, dependent on the result, a secondary screening. The primary screening assesses the presence of recent weight loss, body mass index < 20.5, decreased dietary intake in the preceding week and severe disease. In the secondary screening, the overall score comprises an A-score for nutritional status, a B-score for disease severity and an extra point if aged 70 or above. A higher score indicates greater nutritional risk [26]. Hence, questions on recent changes in body weight and dietary intake will be included in the questionnaire.

##### The scored patient-generated subjective global assessment short form

The PG-SGA SF is a one-page instrument that assesses nutritional risk and nutritional deficit [27]. It includes questions on weight changes, changes in dietary intake (amount or consistency), nutrition impact symptoms and performance status [27]. The PG-SGA SF score ranges from 0 to 36, and a higher score indicates a higher risk of malnutrition. The Danish version has been translated, cross-culturally adapted, and linguistically validated [74]; and is used with permission.

##### M. D. Anderson dysphagia inventory

The MDADI is a self-administered questionnaire on dysphagia-specific QOL in patients with HNC [28]. The Danish version has been translated and culturally adapted, and has been found reliable in terms of internal consistency and test–retest reproducibility [75]. The original version of MDADI consists of 20 items. One item covers overall QOL whereas remaining 19 items form three subdomains: emotional, functional and physical. In addition to a score for each subdomain, a composite score is calculated for the 19 items. The scales range from 20 to 100, and a high score indicates a high degree of functioning. The Danish version contains four additional items concerning specific mechanisms that affect deglutition [75].

#### Rehabilitation needs measured by the REHPA scale

As described under ‘Inclusion and randomisation’, the REHPA Scale is a numerical score of how close or far an individual is from living the life they desire after their disease. The scale ranges from 1 to 9, and a higher score indicate greater rehabilitation needs. In addition to the numerical score, the participant can mark the challenges that prevent them from achieving their goals. Challenges listed in the questionnaire include different practical problems, work-related problems, family problems, physical symptoms, psychological problems and existential problems.

#### Primary outcome

The primary outcome is percentage change in body weight from baseline to 3-month follow-up. Body weight will be measured to the nearest 0.1 kg on calibrated Seca 877/878 scales. In accordance with National Institute for Health Research Southampton Biomedical Research Centre Procedure for Measuring Adult Weight [76], body weight measurements will be continued until three consecutive measurements within 100 g of each other are obtained. The mean of the three will be used in the analyses. Participants will be asked to limit their food and fluid intake two hours before the weighing and to empty their bladder immediately before. For each participant, body weight measurements will be performed at the same time of day (before/after noon) at baseline and 3-month follow-up.

#### Secondary outcomes: patient reported outcome measures

##### Health-related quality of life

Health-related QOL will be measured using the Danish translations of the EuroQol 5D-5 L (EQ-5D-5 L) [77], the EORTC QLQ-C30 [38, 78], and the diagnosis specific EORTC QLQ-H&N35 [78, 79].

The EQ-5D-5 L covers the dimensions mobility, self-care, usual activities, pain/discomfort and anxiety/depression, and a low score indicates a high level of functioning in the given dimension. Overall health is measured with an index score based on the five dimensions and by visual analogue scale (VAS). The index score ranges from − 0.624-1.0 and the VAS scale ranges from 0 to 100. A higher score represents a better self-rated health [77].

Participants’ scores in QLQ-C30 and QLQ-H&N35 will be calculated according to the manual [80]. The tools comprise one global QOL scale, five functional scales and 27 symptom scales. All scales range from 0 to 100, and a high score represents a higher response level. Thus, a high score for a functional scale or global QOL represents a high level of functioning/QOL whereas a high score on a symptom scale represents a high level of symptoms.

##### Symptoms of anxiety and depression

Symptoms of anxiety and depression will be measured with the Danish translation of the Hospital Anxiety and Depression Scale. The scale consists of two subscales for anxiety and depression. The subscales range from 0 to 21, and a high score indicates a high symptom level [81].

#### Secondary outcomes: physical measurements and physical performance tests

##### Body mass index

Body mass index will be calculated as body weight (kg) divided by squared height (m). Height will be measured to the nearest 0.5 cm using a Seca 222 stadiometer.

##### Maximal mouth opening

To assess trismus, maximal mouth opening will be measured in mm using a TheraBite® Range-Of-Motion ROM Scale. Participants will be seated on a chair during the test. The notch of the scale will be placed on the left lower front tooth, and the participant will be asked to open the mouth as widely as possible without discomfort. While still touching the lower front tooth, the scale will be rotated until it also touches the left upper front teeth, and the measuring point will be registered. Three measurements will be performed, and the highest measurement will be used for data analyses.

##### Hand grip strength

Hand grip strength will be measured in kg using a calibrated Jamar hand dynamometer. The measurement protocol is based on recommendations from Roberts et al. [82]. Measurements will be made with the hand dynamometer in the second handle position. Three consecutive measurements in each hand will be performed, and the highest measurement for each hand will be used for data analyses.

##### 30-second chair stand test

The 30-second chair stand test assesses lower body strength [83]. It measures the number of times a person can sit and rise to full standing position from a chair in 30 s. The test protocol follows the method described by Jones et al. [83]. The participant will be instructed to be fully seated between the stands and encouraged to complete as many full stands as possible during the 30 s without using their hands. The final score will be the total number of stands executed correctly. If participants are unable to rise without using their hands, it will be registered that the test is completed in a modified version.

##### 6-minute walk test

The 6-minute walk test is considered a measure of the submaximal level of functional capacity [84].

The test will be performed on a 30-m walking course. Participants will be instructed to walk as many laps as possible during the six minutes without jogging or running. Each minute, the tester will inform the participant about the remaining time, but otherwise the test will be performed in silence. After six minutes, the participant will be asked to stop, and completed distance of the final lap will be measured to the nearest metre. The score will be the total distance walked in metres.

### Sample size

The sample size calculation is based on quantitative data from the previous pilot study [4]. The mean weight change in percent was 1.74 ± 2.37 when restricting to participants with cancer of the pharynx, larynx, or oral cavity and who had completed radiation therapy 1–5 years prior to participation. Based on these data, 30 participants are required in each group to achieve a power of 80% and a significance level of 5%. Thus with an estimated withdrawal rate of 15% [4], we will include 36 participants in each group.

### Data analysis

The statistical analysis plan for the trial is shown in Additional file 5. Data will be analysed in SAS® Enterprise Guide® 7.1 by both per protocol and intention-to-treat principle [85]. Data analyses will not be commenced until all data collection is completed. A blinded researcher (TBM) will analyse the data, and the project group will interpret results before unblinding. Development in outcome scores from baseline to 3-month follow-up will be calculated for each participant, and differences between intervention group and wait-list control group will be tested using a two-sample two-sided t-test for normally distributed data and Mann-Whitney U test for non-normally distributed data. A significance level of 5% will be applied. Effect size will be estimated with Cohens d [86]. Multiple linear regression will be used to assess the influence of potential confounding variables (e.g. time interval from completion of treatment) on intervention effect. Mean baseline values for outcome scores in both groups will be presented in result tables. Simple linear regression will be used to test correlations between developments in outcome scores and baseline scores in NRS 2002, MDADI or PG-SGA SF. Sensitivity, specificity and predictive values of different cut-offs in NRS 2002, MDADI or PG-SGA SF at baseline in relation to a clinically relevant improvement in outcome scores during participation in the programme will be assessed. To avoid missing data, participants who drop out of the trial will be encouraged to participate in follow-up measurements. The percentage and patterns of missing values in outcome variables will be examined. If data are missing at random and the percentage of missing data is not substantial [87], multiple imputation techniques will be used in the intention-to-treat analyses.

### Patient and public involvement

Patients have been involved in several steps of the trial development. A pilot study was conducted [4], where participants through focus groups contributed with ideas for further qualification of the intervention. Furthermore, they contributed to the selection of the nutrition screening and assessment tools for this trial. The preliminary trial protocol was presented at a workshop for REHPA’s user panel. The panel consists of former participants in REHPA’s programmes and representatives from patient organisations. The discussion at the workshop focused on the intervention and on pros and cons of including participants’ relatives. Input from patient involvement led to adjustments of the programme including possibility for counselling with a speech pathologist, optional session with a sexologist, optional session with vocational counselling, and adjustment of breaks during the days. Furthermore, it was decided that the intervention in the present trial will not be aimed at or include relatives, since patients were concerned that it would affect social interaction and candidness among participants.

When the trial is completed, participants will be invited to a symposium with presentation of the main results. Participants will be welcome suggest secondary explorative analyses of patient interest to inform future research questions based on the findings of the NUTRI-HAB trial.

## Ethics and dissemination

The trial will be conducted in accordance with the Declaration of Helsinki [88]. Informed written consent will be obtained from all participants before inclusion. Participants will be informed verbally and in writing that participation is voluntary, and that they can withdraw their consent at any time. Participants will receive no payment for their participation, and their only expenses associated with participation will be transportation costs to the rehabilitation centre. For ethical reasons, we will use a wait-list control group, who also receives the intervention after 3-month follow-up. Based on the prior pilot study [4] it is expected that participants will benefit from participation, and there are no expected risks associated with participation. The Regional Committees on Health Research Ethics for Southern Denmark have assessed the duty to notify for the present trial (journal number 20182000–165). Based on Danish legislation, the committees concluded that the trial is not subject to the duty to notify since no biological material is included. The trial is registered by The Danish Data Protection Agency, registration number 2012-58-0018, approval number 18/14847, and registered in the database Clinical Trials (www.clinicaltrials.gov, NCT03909256). Amendments to the protocol will be made public at clinicaltrials.gov.

Results will be published in international peer-reviewed journals and presented at national and international conferences.

Within the confines of Danish legislation, anonymised data from the trial will be available for other researchers upon reasonable request when results have been published.

## Discussion

This is the first randomised controlled trial to test the effect of a multidisciplinary residential nutritional rehabilitation programme in patients treated for HNC. While residential rehabilitation programmes may be beneficial for patients treated for HNC, the residential rehabilitation programme in this trial is also intensive and requires participants to be self-reliant and to participate actively. Hence, the residential rehabilitation programme is distinct from typical inpatient rehabilitation services, and participants may be of better health than in other inpatient rehabilitation services. Requiring participants to be self-reliant may exclude the most vulnerable patients from participating and pose a risk of selection bias. In the present trial, recruitment through a nationwide survey gives a unique possibility to describe the trial population and to identify potential selection bias.

Additional methodological strengths of the trial include randomisation, blinded data analysis and blinded interpretation of results. The use of a wait-list control group may enhance trial adherence, and it meets the ethical challenges of using a non-intervention control group. However, the improvement typically seen among individuals in a non-intervention control group tend to be smaller among individuals in a wait-list control group. Hence, concerns have been raised that trials using wait-list control groups may overestimate the effect of intervention [89]. This will be considered when interpreting the results.

Multidisciplinary residential rehabilitation programmes are resource-intensive, and they may not be readily implementable in existing municipal or community-based rehabilitation services everywhere. This may affect the applicability of the trial results. However, establishing residential rehabilitation programmes across municipalities or institutions could allow for offering group-based diagnosis specific rehabilitation services even in small municipalities or communities with few patients treated for HNC. Hence, this trial will serve as a proof-of-concept trial, while future studies on the potential implementation of residential rehabilitation services in existing health services may be relevant depending on trial results.

As the trial will explore the potential of different nutrition screening and assessment tools in the assessment of rehabilitation needs in patients treated for HNC, the trial will create knowledge about how selection and prioritisation of nutritional rehabilitation aimed at patients treated for HNC should be offered. The results may contribute to a better organisation and use of existing resources in benefit of patients treated for HNC.

## Supplementary information

**Additional file 1.** Mean body weight and quality of life scores at baseline and 3- month follow-up in patients treated for head and neck cancer who participated in the pilot study of the intervention.

**Additional file 2.** SPIRIT checklist.

**Additional file 3.** Overview and description of physical and informational materials used in the NUTRI-HAB trial.

**Additional file 4.** Course programme for the initial five days and the two days follow-up of the multidisciplinary residential nutritional rehabilitation programme in the NUTRI-HAB trial.

**Additional file 5.** Statistical analysis plan for the NUTRI-HAB trial.

## Data Availability

Within the confines of Danish legislation, the anonymised data from the trial will be available for other researchers upon reasonable request when results have been published.

## Abbreviations

EORTC: The European Organization for Research and Treatment of Cancer
HNC: head and neck cancer
MDADI: M. D. Anderson Dysphagia Inventory
NRS 2002: Nutritional Risk Screening 2002
PG-SGA SF: The Scored Patient-Generated Subjective Global Assessment Short Form
QOL: Quality of life
REDCap: Research Electronic Data Capture
