# Supplementary material for: Rationale and design of a randomised controlled trial investigating the effect of multidisciplinary nutritional rehabilitation for patients treated for head and neck cancer (the NUTRI-HAB trial)
Source: Nutr J. 2020 Mar 17;19:21. doi: 10.1186/s12937-020-00539-7 (PMC7079410; doi:10.1186/s12937-020-00539-7)
Supplement: Supplementary file 1 — Additional file 1. Mean body weight and quality of life scores at baseline and 3- month follow-up in patients treated for head and neck cancer who participated in the pilot study of the intervention. [file 12937_2020_539_MOESM1_ESM.pdf]

Additional file 1: Body weight and health related quality of life (QOL) at baseline and at 3-month follow-up in Danish patients treated for head and neck cancer who participated in a multidisciplinary residential nutritional rehabilitation programme[a].

	Baseline	3-month follow-up	p-value[b]
**Body weight (n=34)**	65.0 ±15.1	66.0 ±15.5	0.042*
**EORTC QLQ-C30**			
**Global health status/QOL (n=32)**	59.4 ±22.7	58.1 ±19.8	0.558
**Functional scales**			
- Physical functioning (n=32)	77.5 ±16.7	82.7 ±13.2	0.038*
- Role functioning (n=32)	68.8 ±26.4	72.9 ±27.7	0.312
- Emotional functioning (n=32)	65.4 ±21.6	71.9 ±22.7	0.154
- Cognitive functioning (n=32)	72.4 ±21.8	75.0 ±25.8	0.233
- Social functioning (n=31)	68.8 ±25.0	76.9 ±16.7	0.210
**Symptom scales/items**			
- Fatigue (n=32)	42.0 ±23.2	41.3 ±25.1	0.719
- Nausea and vomiting (n=32)	13.0 ±18.8	7.3 ±11.9	0.066
- Pain (n=32)	25.5 ±26.1	28.1 ±27.3	0.382
- Dyspnoea (n=32)	20.8 ±30.2	15.6 ±25.4	0.195
- Insomnia (n=32)	36.5 ±27.3	32.3 ±32.2	0.194
- Appetite loss (n=31)	47.3 ±35.3	36.6 ±31.5	0.054
- Constipation (n=32)	14.6 ±22.3	11.5 ±16.1	0.366
- Diarrhoea (n=32)	14.6 ±20.6	11.5 ±23.4	0.432
- Financial difficulties (n=32)	10.4 ±17.9	11.5 ±24.8	0.948
**EORTC QLQ-H&N35**			
**Symptom scales/items**			
- Pain (n=31)	31.7 ±22.8	28.2 ±24.4	0.760
- Swallowing (n=30)	35.8 ±23.4	30.0 ±24.3	0.034*
- Sensory problems (n=31)	35.5 ±25.7	30.6 ±25.1	0.059
- Speech problems (n=31)	22.2 ±21.1	15.4 ±16.4	0.016*
- Trouble with social eating (n=30)	47.8 ±31.3	38.4 ±27.3	0.010*
- Trouble with social contact (n=30)	12.9 ±15.4	11.8 ±17.1	0.508
- Less sexuality (n=26)	37.2 ±38.7	33.3 ±36.2	0.873
- Teeth (n=30)	42.2 ±36.0	41.1 ±39.8	0.873
- Opening mouth (n=30)	25.6 ±32.4	27.8 ±35.1	0.964
- Dry mouth (n=31)	73.1 ±29.1	71.0 ±30.7	0.629
- Sticky saliva (n=30)	51.1 ±36.9	50.0 ±35.8	0.719
- Coughing (n=31)	31.2 ±29.7	31.2 ±33.3	0.992
- Felt ill (n=31)	21.5 ±20.3	23.7 ±27.5	0.392
- Pain killers (n=31)	51.6 ±50.8	38.7 ±49.5	0.103
- Nutritional supplements (n=30)	33.3 ±47.9	40.0 ±49.8	0.527
- Feeding tube (n=28)	25.0 ±44.1	10.7 ±31.5	0.046*
- Weight loss (n=29)	41.4 ±50.1	20.7 ±41.2	0.014*
- Weight gain (n=27)	22.2 ±42.4	33.3 ±48.0	0.257

[a] Only participants with both a baseline and a follow-up measurement of the given variable were included in the analyses and thus in the table. The included numbers of participants are presented for each variable.
[b] Differences between body weight at baseline and follow up were tested using a two-sided paired t-test while differences between QOL scores at baseline and follow-up were tested using the Wilcoxon sign rank test.
$^{*}p<0.05$

Data are presented as mean values and standard deviations. The European Organization for Research and Treatment of Cancer's (EORTC) QLQ-C30 and QLQ-H&N35 were used to assess QOL. All scales and single-item measures range in score from 0 to 100. A high score represents a higher response level. Thus a high score for a functional scale or global QOL represents a high level of functioning/QOL whereas a high score on a symptom scale represents a high level of symptoms.
