# Supplementary material for: Rationale and design of a randomised controlled trial investigating the effect of multidisciplinary nutritional rehabilitation for patients treated for head and neck cancer (the NUTRI-HAB trial)
Source: Nutr J. 2020 Mar 17;19:21. doi: 10.1186/s12937-020-00539-7 (PMC7079410; doi:10.1186/s12937-020-00539-7)
Supplement: Supplementary file 3 — Additional file 3. Overview and description of physical and informational materials used in the NUTRI-HAB trial. [file 12937_2020_539_MOESM3_ESM.pdf]

Additional file 3: Overview and description of physical and informational materials used in the NUTRI-HAB Trial.

Trial materials (with the exception of copyrighted validated questionnaires) are available upon request to the corresponding author (mabk@kp.dk). All trial materials are in Danish.

	Description of material(s)	***How will the material(s) be distributed/administered?***
**MATERIALS FOR PARTICIPANT RECRUITMENT, INCLUSION AND RETENTION**		
- Invitation letter	1-page invitation letter with information on the trial and the dates on which the individual recipient is invited to participate in the programme, the follow-up and the outcome measurements in the regional outpatient clinics.	Sent electronically to e-Boks[a] or through postal mail to individuals on the invitation lists for intervention group or wait-list control group.
- Programme leaflet	4-page leaflet with information on the multidisciplinary residential nutritional rehabilitation programme.	Sent together with 'Invitation letter'.
- Consent form for participating in the NUTRI-HAB Trial	3-page form with 2 pages of information regarding participation in the trial and trial registration details and 1 page to be signed by the participant and the researcher.	Handed out to participants (and signed form collected with copy provided to participant) on the first day of the residential stay for the intervention group, and on the day of the outcome measurements in the outpatient clinics for the control group.
- Consent form for registration in the rehabilitation centre's clinical research database	3-page form with 2 pages of information regarding the rehabilitation centre's clinical research database and 1 page to be signed by the participant and the researcher.	Handed out to participants (and signed form collected with copy provided to participant) on the first day of the residential stay for the intervention group, and on the day of the outcome measurement in the outpatient clinics for the control group.
- Welcome letter (five days residential stay)	1-page letter with practical information on arrival etc.	Sent to individuals who accepted invitation to participate. Sent electronically to e-Boks or through postal mail prior to the five days residential stay.

- Leaflet about the rehabilitation centre	4-page folder with practical information about the residential rehabilitation centre.	Sent to participants together with 'Welcome letter (five days residential stay)'.
- Welcome letter (two days follow-up residential stay)	1-page letter with practical information on arrival etc.	Sent electronically to e-Boks or through postal mail prior to the two days follow-up residential stay.
- Invitation for outcome measurements in the regional outpatient clinics	1-page letter with information on date, time, and place for the outcome measurement, and instructions to wear comfortable clothes and to limit food and fluid intake the last two hours prior to the measurements.	Sent electronically to e-Boks or through postal mail prior to the outcome measurement in the outpatient clinics.
**INTERVENTION MATERIALS**		
- Course programme (five days residential stay)	An overall course programme (not including individual activities)	Sent to participants together with 'Welcome letter (five days residential stay)'.
- Course programme (two days follow-up residential stay)	An overall course programme (not including individual activities)	Sent to participants together with 'Welcome letter (two days follow-up residential stay)'.
- Individualised course programme (five days residential stay)	A detailed individualised course programme with scheduled individual activities and selected optional sessions.	Handed out to participants on the first day of the five days residential stay.
- Individualised course programme (two days follow-up residential stay)	A detailed individualised course programme with scheduled individual activities and selected optional sessions.	Handed out to participants on the first day of the two days follow-up residential stay.
- 'Participant book'	The participant book includes a training manual with 16 different swallowing exercises, a training dairy where participants can register their training, space for taking notes throughout the programme, and dates for telephone consultations with the clinical dietitian.	Handed out to participants on the five days residential stay immediately before the session with 'Swallowing exercises'.

**Materials from the different patient education sessions in the programme**		
- Materials from 'Theoretical session on eating problems'	Handouts of the PowerPoint slides used in the session.	Sent to participants after the five days residential stay.
- Materials from the 'Kitchen workshop'	A recipe book with 45 different recipes and information about the five basic tastes and how they complement each other. An apron with the Danish name of the programme and logos of the collaborating institutions printed on it.	Recipe book: Handed out in the session.<br>Apron: Handed out in the session for intervention group and handed out on the day of the outcome measurements in the outpatient clinics for control group.
- Materials from the session 'Swallowing exercises'	Handouts of the PowerPoint slides used in the session.	Sent to participants after the five days residential stay.
- Materials from the session 'Dental problems and oral hygiene'	Handouts of the PowerPoint slides used in the session and a 2-page summary with take-home messages.	Sent to participants after the five days residential stay.
- Materials from the session on 'Psychological reactions to cancer'	Handouts of the PowerPoint slides used in the session.	Sent to participants after the five days residential stay.
- Materials from the session on 'Fatigue and sleep problems'	Handouts of the PowerPoint slides used in the session.	Sent to participants after the five days residential stay.
- Materials from the session on 'Motivation, goal setting and action plans'	Handouts of the PowerPoint slides used in the session. A 2-page template for the participants' action plans.	The action plan template: Handed out in the session.<br>The handouts of PowerPoint slides: Sent to participants after the five days residential stay.

**DATA COLLECTION FORMS**<br>**Forms/questionnaires to be filled out by participants**		
- Questionnaire with outcome measures	A 117-item questionnaire comprising EQ-5D-5L, EORTC QLQ-C30, EORTC QLQ-H&N35, PG-SGA SF, MDADI, HADS, and questions on body weight history, dietary intake, civil status, educational level, and occupational status.	Sent electronically to e-Boks one week prior to the five days residential stay, the two days follow-up residential stay, and the outcome measurements in the outpatient clinics. Participants without e-Boks: Possibility to fill out electronically or in paper on the location of the measurement.
- Questionnaire with information prior to participation in the five days residential stay	Questionnaire with practical information required by the rehabilitation centre: specific dietary requirements, comorbidities, emergency contact, expectations for the programme, specific challenges they want to address, and which optional activities they want to participate in.	Sent electronically to e-Boks or through postal mail together with 'Welcome letter (five days residential stay)'.
- Questionnaire with information prior to participation in the two days follow-up residential stay	Questionnaire with practical information required by the rehabilitation centre: specific dietary requirements, expectations for the follow-up, and which optional activities they want to participate in.	Sent electronically to e-Boks or through postal mail together with 'Welcome letter (two days follow-up residential stay)'.
- Evaluation form (five days residential stay)	1-page evaluation form where participants will be asked to evaluate the overall residential stay, the different sessions and indicate whether they participated in the specific session.	Handed out to participants on the first day of the five days residential stay and collected from participants on the last day.
- Evaluation form (two days follow-up residential stay)	1-page evaluation form where participants will be asked to evaluate the overall residential stay, the different sessions and indicate whether they participated in the specific session.	Handed out to participants on the first day of the two days follow-up residential stay and collected from participants on the last day.

**Data collection forms to be used by health professionals**		
- Form to enter results from physical test and measurements	1-page standardised form to register measured weight, height, maximal mouth opening, hand grip strength, 30-second chair stand test, 6-minute walk test, and initials of the physiotherapist performing the measurements.	Used by physiotherapists at the five days residential stay, the two days follow-up residential stay, and the outcome measurements in the outpatient clinics.
- Standardised form for the individual counselling with clinical dietitian (five days residential stay – intervention group)	2-page form with space for summary of relevant outcome measurements, participant's evaluation of own weight, and a summary of the individual counselling.	Used by dietitians at the five days residential stay.
- Standardised form for the individual counselling with clinical dietitian (five days residential stay – control group)	2-page form with space for summary of relevant outcome measurements, participant's evaluation of own weight, changes in health status, participation in other rehabilitation services, and a summary of the individual counselling.	Used by dietitians at the five days residential stay.
- Standardised form for summary of the telephone consultations with clinical dietitian	1-page form with space for summary of the two telephone consultations.	Used by dietitians for the telephone consultations between the five days residential stay and the two days follow-up residential stay.
- Standardised form for the individual counselling with clinical dietitian (two days follow-up residential stay)	2-page form with space for summary of previous and current counselling, relevant outcome measurements, changes in health status, participation in other rehabilitation services, and experiences of participating in the programme.	Used by dietitians at the two days follow-up residential stay.

[a] e-Boks is a secure digital mailbox linked to the individual's civil registration number. In Denmark, it is mandatory to have e-Boks unless a citizen applies for exemption.

EORTC: European Organization for Research and Treatment of Cancer, PG-SGA SF: The Scored Patient Generated Subjective Global Assessment Short Form, HADS: Hospital Anxiety and Depression Scale, MDADI: M. D. Anderson Dysphagia Inventory.
