# Supplementary material for: Rationale and design of a randomised controlled trial investigating the effect of multidisciplinary nutritional rehabilitation for patients treated for head and neck cancer (the NUTRI-HAB trial)
Source: Nutr J. 2020 Mar 17;19:21. doi: 10.1186/s12937-020-00539-7 (PMC7079410; doi:10.1186/s12937-020-00539-7)
Supplement: Supplementary file 4 — Additional file 4. Course programme for the initial five days and the two days follow-up of the multidisciplinary residential nutritional rehabilitation programme in the NUTRI-HAB trial. [file 12937_2020_539_MOESM4_ESM.pdf]

*Kristensen MB, Wessel I, Beck AM, Dieperink KB, Mikkelsen TB, Møller JJK, Zwisler AD. Rationale and design of a randomised controlled trial investigating the effect of multidisciplinary nutritional rehabilitation for patients treated for head and neck cancer (the NUTRI-HAB Trial), Nutrition Journal*

**Additional file 4:** Example of the course programme for the initial five days and the two days follow-up of the multidisciplinary residential nutritional rehabilitation programme in the NUTRI-HAB Trial.

| INITIAL RESIDENTIAL STAY | | | | | FOLLOW-UP RESIDENTIAL STAY AFTER 3 MONTHS | |
|---|---|---|---|---|---|---|
| **DAY 1** | **DAY 2** | **DAY 3** | **DAY 4** | **DAY 5** | **DAY 1** | **DAY 2** |
| | BREAKFAST BUFFET | BREAKFAST BUFFET | BREAKFAST BUFFET | BREAKFAST BUFFET | | BREAKFAST BUFFET |
| | MORNING ASSEMBLY | MORNING ASSEMBLY | MORNING ASSEMBLY | MORNING ASSEMBLY | | MORNING ASSEMBLY |
| Arrival<br><br>Welcome session with presentation of the programme<br>(course leader and clinical dietitian)<br><br>Walk and talk | Practical kitchen workshop<br>(clinical dietitian) | Psychological reactions to cancer<br>(psychologist) | Physical activity<br>(physiotherapist)<br><br>Optional session:<br>Fatigue and sleep problems (nurse)<br>*OR*<br>Vocational counselling<br>(social worker) | Motivation, goal setting and action plans<br>(social worker and course leader)<br><br>Individual work and group discussion on action plans<br>(social worker and course leader) | Arrival<br><br>Welcome and presentation of the program<br>(course leader and clinical dietitian)<br><br>What's new within the last three months?<br>(course leader and clinical dietitian) | Physical activity<br>(physiotherapist)<br><br>Optional session:<br>Sexuality and intimacy<br>(sexologist)<br>*OR*<br>Meaning and values in life<br>(psychologist) |
| LUNCH BUFFET | LUNCH BUFFET | LUNCH BUFFET | LUNCH BUFFET | LUNCH BUFFET | LUNCH BUFFET | LUNCH BUFFET |
| Introduction round<br>(course leader and central health professionals)<br><br>Theoretical session on eating problems<br>(clinical dietitian) | DATA COLLECTION<br>Physical tests<br>(physiotherapist)<br><br>Yoga<br>(physiotherapist) | Swallowing exercises<br>(occupational therapist)<br><br>Individual dietary counselling<br>(clinical dietitian) | Dental problems and oral hygiene<br>(dental hygienist)<br><br>Individual counselling<br>(depending on the participant's needs)<br><br>Massage therapy<br>(massage therapist) | Closing session and farewell<br>(course leader and clinical dietitian) | DATA COLLECTION<br>Physical tests<br>(physiotherapist)<br><br>Individual dietary counselling (clinical dietitian) | Closing session and farewell<br>(course leader and clinical dietitian) |
| DINNER | DINNER | DINNER | DINNER | | DINNER | |
| Social activity | Group conversation on existence<br>(priest) | (Possibility to go for a walk, watch movies, play games etc.) | | | | |
