# Supplementary material for: Rationale and design of a randomised controlled trial investigating the effect of multidisciplinary nutritional rehabilitation for patients treated for head and neck cancer (the NUTRI-HAB trial)
Source: Nutr J. 2020 Mar 17;19:21. doi: 10.1186/s12937-020-00539-7 (PMC7079410; doi:10.1186/s12937-020-00539-7)
Supplement: Supplementary file 5 — Additional file 5. Statistical analysis plan for the NUTRI-HAB trial. [file 12937_2020_539_MOESM5_ESM.pdf]

Additional file 5: Statistical analysis plan (SAP) for The NUTRI-HAB trial.

**ADMINISTRATIVE INFORMATION**	
Title	Statistical analysis plan (SAP) for a randomised controlled trial investigating the effect of multidisciplinary nutritional rehabilitation for patients treated for head and neck cancer (the NUTRI-HAB Trial)
Trial registration details	• ClinicalTrials.gov Identifier: NCT03909256<br>• The Danish Data Protection Agency: registration number 2012-58-0018, approval number 18/14847<br>• The Regional Committees on Health Research Ethics for Southern Denmark: journal number 20182000-165
Principal investigator	Marianne Boll Kristensen, PhD Fellow, RD,<br>*REHPA, The Danish Knowledge Centre for Rehabilitation and Palliative Care & Department of Nursing and Nutrition, University College Copenhagen &*<br>*OPEN, Odense Patient data Explorative Network, Odense University Hospital*
Data analyst	Tina Broby Mikkelsen, PhD,<br>*REHPA, The Danish Knowledge Centre for Rehabilitation and Palliative Care*
SAP developed by	Tina Broby Mikkelsen, Marianne Boll Kristensen.
SAP version date	15/10-2019 *(Minor revisions based on reviewers' comments carried out 5/2-2020)*
SAP version approval	SAP version approved by the project group 28/10-2019.
**TRIAL INFORMATION**	
Objectives	The objectives of the trial are:<br>• To test the effect of a multidisciplinary residential nutritional rehabilitation programme compared to standard care on the primary outcome body weight and secondary outcomes health-related quality of life, physical function and symptoms of anxiety and depression in patients curatively treated for head and neck cancer<br>• To test whether a potential effect of a multidisciplinary residential nutritional rehabilitation programme is associated with the participants' nutritional status, nutritional risk or presence of nutrition impact symptoms measured by measured by Nutritional Risk Screening 2002 (NRS 2002)[1], the Scored Patient-Generated Subjective Global Assessment Short Form (PG-SGA SF)[2], and the M. D. Anderson Dysphagia Inventory (MDADI)[3] at entry to the programme.
Trial design	The trial is a randomised controlled trial with recruitment through a nationwide survey. Participants will be randomised into either intervention group or a wait-list control group in a 1:1 allocation ratio.
Trial setting	The trial will be carried out at REHPA, the Danish Knowledge Centre for Rehabilitation and Palliative Care in Nyborg, Denmark between May 2019 and December 2019
Participants	Participants will be recruited among respondents of a nationwide survey in Danish patients treated for head and neck cancer. The following inclusion criteria apply:<br>*Register-based information*<br>• Have been diagnosed with cancer of the larynx, pharynx, or oral cavity<br>• Have completed curatively intended treatment with radiation therapy 1-5 years before survey distribution (1st of March 2014 to 28th of February 2018)<br>• Are aged ≥ 18 years<br>*Self-reported information collected through the survey*<br>• Have no active head and neck cancer or any other active cancer at the time for completion of the survey

	• Are self-reliant<br>• Are able to speak and understand Danish<br>• Are willing to participate in a multidisciplinary residential nutritional rehabilitation programme
Sample size	The sample size calculation is based on quantitative data from a previous pilot study[4] and an expectation to see a difference of 1.74±2.37 in primary outcome (weight change in percent) between groups. 30 participants are required in each group to achieve a power of 80% and a significance level of 5%. Thus with an estimated withdrawal rate of 15%, 36 participants will be included in each group.
Randomisation and inclusion	Individuals who meet the inclusion criteria will be randomised into invitation lists for intervention group or wait-list control group. The allocation ratio will be 1:1, and allocated individuals will be placed in random order on the numbered invitation list. Individuals will be invited for participation in the order they appear on the given invitation list.<br><br>Data analyst, who is not involved in the study intervention or outcome assessment, will randomise participants in STATA/IC 15.1.
Blinding	Data analysis will be blinded. A trial-independent researcher codes the data set prior to analyses, and the code will be kept in a sealed envelope. The project group will interpret the blinded results before unblinding.
**TIMELINE OF THE TRIAL AND DATA ANALYSES**	
Trial timeline	The trial is expected to run from May 2019 to early December 2019.<br>For half of the intervention group and half of the wait-list control group, baseline measurements will be in May 2019, and 6-month follow-up measurements will be late October 2019. For the other half of the participants, baseline measurements will be in June 2019, and 6-month follow-up measurements will be late November 2019.
Timeline for data collection	Data is collected at baseline, 3-month follow-up and 6-month follow up.<br>The last physical measurements are planned to late November 2019 (27/11-2019). As an effort to minimise missing data, data collection will not be considered completed until early December 2019 (09/12-2019) allowing for eight extra work days to obtain data from potential no-show participants.
Timeline for data preparation and analysis	The data set will be prepared immediately after data collection is considered completed and will be sent to data analyst mid December 2019 (16/12-2019).<br>Data analyses will be completed no later than early January 2020 (07/01-2019), where the blinded results will be interpreted by the project group.
**GENERAL STATISTICAL CONSIDERATIONS**	
Significance level	A significance level of 5% will be applied.
Protocol violations and exclusions from the trial	Protocol deviations and exclusions from the trial (including reasons for exclusion) will be reported for each group of the trial.<br><br>Data on primary and secondary outcomes will be analysed by both the intention-to-treat principle and per protocol, and results from both types of analysis will be presented in publications.<br><br>In analyses by the intention-to-treat principle, all participants will be analysed in the trial group to which they were randomised even if they do not receive the allocated treatment. In the per protocol analyses, only participants who received the allocated treatment is included.

<table>
<tr><td>Missing data</td><td>All efforts will be made to minimise missing data. Participants who drop out of the trial will be encouraged to participate in follow-up measurements and to complete follow-up questionnaires.

In the event of missing data, the percentage and patterns of missing values in outcome variables will be examined. If data are missing at random and the percentage of missing data is not substantial[5], multiple imputation techniques will be used in the intention-to-treat analyses.</td></tr>
<tr><td>Statistical software</td><td>Data will be analysed in SAS® Enterprise Guide® 7.1</td></tr>
<tr><td colspan="2">DATA PREPARATION</td></tr>
<tr><td>Data entry</td><td>Patient reported data will primarily be collected through electronic questionnaires distributed through REDCap. In the event that participants fill out a paper-based questionnaire instead, the data will be entered in REDCap by one researcher, and the entered data will be double-checked by a second researcher.</td></tr>
<tr><td>Preparation of data set</td><td>Principal investigator will prepare the data set and remove all possible identifiers including dates and time stamps.</td></tr>
<tr><td>Coding</td><td>A trial-independent researcher will code the prepared data set and put the code in a sealed envelope.</td></tr>
<tr><td colspan="2">DATA ANALYSES</td></tr>
<tr><td>Descriptive statistics</td><td>Descriptive statistics will be used for baseline characteristics of participants in the intervention and wait-list control group. The following variables will be included in the presentation of baseline characteristics:
• Age
• Gender
• Cancer diagnosis (pharynx, larynx, oral cavity)
• Time (months) interval since completion of radiation therapy
• Civil status
• Educational level
• Occupational status
• Rehabilitation needs measured by the REHPA scale (≥3 point, <3 point)

Numerical variables will be presented as median [range] or mean (SD) and categorical variables as number of participants (%).</td></tr>
<tr><td>Assessment of selection bias</td><td>To assess potential selection bias, it will be tested whether the trial population differ from the remaining survey population (including non-responders) with regards to the following variable: age, gender, cancer diagnosis and time interval since completion of radiation therapy.</td></tr>
<tr><td>Analysis of primary outcome</td><td>The primary outcome is percent change in body weight from baseline to 3-month follow-up.

Differences between group means will be tested using a two-sample two-sided t-test, and effect size will be estimated with Cohens d[6].
An estimate of the difference between groups along with a 95% CI and a two-sided p value for the null hypothesis of no difference between groups will be reported.

The mean body weight in each group at the different time points will be presented in publications.</td></tr>
</table>

	Multiple linear regression will be used to assess the influence of potential confounding variables (e.g. time interval from completion of treatment) on intervention effect
Analyses of secondary outcome measures	Secondary outcomes include changes from baseline to 3-month follow up in the following outcome measures:<br><br>Patient reported outcome measures:<br>• EQ-5D-5L[7]<br>• EORTC QLQ-C30[8,9]<br>• EORTC QLQ-H&N35[9,10]<br>• Hospital Anxiety and Depression Scale[11]<br><br>Physical test and measurements:<br>• Maximal mouth opening<br>• Hand grip strength<br>• 30-second chair stand test<br>• 6-minute walk test<br><br>All patient-reported outcome measures will be scored according to manuals.<br><br>Differences between group means will be tested using a two-sample two-sided t-test or Mann-Whitney U test depending on distribution of data. Effect size will be estimated with Cohens d[6].<br>For all analyses on secondary outcome, an estimate of the difference between groups along with a 95% CI and a two-sided p value for the null hypothesis of no difference between groups will be reported.<br><br>For all secondary outcomes, the mean score in each group at the different time points will be presented in publications.
Analysis between intervention effect and nutrition screening/assessment scores at baseline and	Linear regression will be used to test potential associations between developments in outcome scores from baseline to 3-month follow-up and baseline scores in NRS 2002, MDADI or PG-SGA SF.<br><br>Sensitivity, specificity and predictive values of different cut-offs in NRS 2002, MDADI or PG-SGA SF at baseline in relation to a clinically relevant improvement in outcome scores during participation in the programme will be assessed.
Planned subgroup analyses	Subgroup analyses will be performed to investigate whether the intervention has different effects on different subgroups of participants e.g. grouped by time from treatment completion.
Exploratory analyses	All data collected from baseline to 6-month follow-up will be used for relevant exploratory analyses including analyses of the long-term effect of the intervention and of whether the selected nutrition screening tools are labile and able to reflect changes over time.
**INTERPRETATION OF RESULTS**	
Interpretation of results	Data analyst will present the blinded results on a meeting with the rest of the project group and involved clinicians. Baseline characteristics and number of participants in each group for the different analyses will not be displayed at the presentation, as these data may reveal the coding of the groups to the clinicians, who delivered the intervention.

	The project group initially interprets the blinded results on the assumption that one specific group is the intervention group, and afterwards on the assumption that the other group is the intervention group. The group writes down the conclusions for both possible scenarios.
Unblinding	When the conclusions for the two possible scenarios have been written down, the principal investigator will open the sealed envelope and reveal the blinding code.

References used in SAP

1. Kondrup J, Ramussen HH, Hamberg O, Stanga Z, Ad Hoc ESPEN Working Group. Nutritional risk screening (NRS 2002): A new method based on an analysis of controlled clinical trials. Clin Nutr. 2003;22(3):321-336. doi:10.1016/S0261-5614(02)00214-5.
2. Jager-Wittenaar H, Ottery FD. Assessing nutritional status in cancer: Role of the Patient-Generated Subjective Global Assessment. Curr Opin Clin Nutr Metab Care. 2017. doi:10.1097/MCO.0000000000000389.
3. Hajdú SF, Plaschke CC, Johansen C, Dalton SO, Wessel I. Cross-Cultural Translation, Adaptation and Reliability of the Danish M. D. Andeson Dysphagia Inventory (MDADI) in Patients with Head and Neck Cancer. *Dysphagia*. 2017;32(4):472-479. doi:10.1007/s00455-017-9785-3.
4. Kristensen MB, Mikkelsen TB, Beck AM, Zwisler AD, Wessel I, Dieperink KB. To eat is to practice – Managing eating problems after head and neck cancer. *J Cancer Surviv*. 2019;13(5):792-803. doi: 10.1007/s11764-019-00798-2.
5. Jakobsen JC, Gluud C, Wetterslev J, Winkel P. When and how should multiple imputation be used for handling missing data in randomised clinical trials - a practical guide with flowcharts. BMC Med Res Methodol. 2017 Dec 6;17(1):162. doi: 10.1186/s12874-017-0442-1.
6. Cohen J. Statistical power analysis for the behavioural sciences. 2. ed. Hillsdale: Lawrence Erlbaum; 1988.
7. Herdman M, Gudex C, Lloyd A, Janssen M, Kind P, Parkin D, et al. Development and preliminary testing of the new five-level version of EQ-5D (EQ-5D-5L). *Qual Life Res*. 2011;20(10):1727-36. doi: 10.1007/s11136-011-9903-x.

8. Aaronson NK, Ahmedzai S, Bergman B, Bullinger M, Cull A, Duez NJ, et al. The European Organization for Research and Treatment of Cancer QLQ-C30: a quality-of-life instrument for use in international clinical trials in oncology. *J Natl Cancer Inst*. 1993 Mar 3;85(5):365-76. doi:10.1093/jnci/85.5.365.
9. Bjordal K, De Graeff A, Fayers PM, Hammerlid E, van Pottelsberghe C, Curran D, et al. A 12 country field study of the EORTC QLQ-C30 (version 3.0) and the head and neck cancer specific module (EORTC QLQ-H&N35) in head and neck patients. *Eur J Cancer*. 2000;36(14):1796-807. doi:10.1016/S0959-8049(00)00186-6.
10. Bjordal K, Ahlner-Elmqvist M, Tollesson E, Jensen AB, Razavi D, Maher EJ, et al. Development of a european organization for Research and treatment of cancer (eortc) questionnaire module to be used in quality of life assessments in head and neck cancer patients. *Acta Oncologica*, 1994;33:8, 879-885, doi: 10.3109/02841869409098450.
11. Zigmond AS, Snaith RP. The Hospital Anxiety and Depression Scale. *Acta Psychiatr Scand*. 1983;67(6):361-70. doi:10.1111/j.1600-0447.1983.tb09716.x.
